# Recognition of mental disorders among a multiracial population in Southeast Asia

**DOI:** 10.1186/s12888-016-0837-2

**Published:** 2016-05-04

**Authors:** Siow Ann Chong, Edimansyah Abdin, Louisa Picco, Shirlene Pang, Anitha Jeyagurunathan, Janhavi Ajit Vaingankar, Kian Woon Kwok, Mythily Subramaniam

**Affiliations:** Research Division, Institute of Mental Health, Buangkok Green Medical Park, 10 Buangkok View, Singapore, 539747 Singapore; Division of Sociology, Nanyang Technological University, Singapore, 637332 Singapore

**Keywords:** Mental health literacy, Help-seeking, Population study, Public beliefs, Vignette

## Abstract

**Background:**

Mental health literacy is an important mediating factor in help-seeking behavior. An important component of this literacy is the proper recognition of mental disorders. The aim of this population-based study in Singapore was to determine the proportion of adults in the resident population who were able to recognize vignettes pertaining to alcohol abuse, dementia, depression, obsessive compulsive disorder (OCD) and schizophrenia correctly. The sociodemographic characteristics that were associated with the ability to correctly recognize these disorders were also examined.

**Methods:**

This was a nationwide cross-sectional study that involved establishing mental health literacy using a vignette approach. Respondents were recruited using a disproportionate stratified sampling design by age and ethnic groups. Face-to face-interviews were conducted with respondents aged 18 to 65 years belonging to Chinese, Malay, Indian and Other ethnic groups.

**Results:**

A total of 3,006 respondents completed the survey (response rate of 71 %). The most well recognized conditions were dementia (66.3 %), alcohol abuse (57.1 %) and depression (55.2 %). The least recognized were OCD (28.7 %) and schizophrenia (11.5 %). Younger age and higher educational levels were found to be significant factors associated with the better recognition of specific disorders.

**Conclusion:**

The relatively high rate of recognition of dementia was likely to be due to the emphasis on public education programmes on dementia which is viewed as an emerging challenge due to Singapore’s rapidly ageing population. The role of education and the portrayal of depression and alcohol related problems in the local mass media are possible influences in their better recognition as compared to OCD and schizophrenia. Sociodemographic characteristics influencing mental health literacy need to be considered in planning intervention strategies that target mental health literacy.

**Electronic supplementary material:**

The online version of this article (doi:10.1186/s12888-016-0837-2) contains supplementary material, which is available to authorized users.

## Background

Mental health disorders are common in the general population: the World Health Organization (WHO) has estimated that over a third of people in most countries meet the criteria for a mental health disorder at some point in their life [1]. An epidemiological study of the resident general population in Singapore - an island city-state in Southeast Asia - showed that 12.0 % of those aged 18 years and above had at least one life-time affective, anxiety, or alcohol use disorder [2]. This nation-wide survey was an intrinsic part of Singapore’s first ever National Mental Health Policy and Blueprint which was implemented in 2007 and sought to identify gaps in the local mental healthcare landscape. The most striking finding of this population survey was the large treatment gap (i.e. the proportion of individuals who require care but do not receive treatment) associated with these mental disorders. The largest treatment gap was among those with alcohol abuse (96.2 %) followed by obsessive compulsive disorder (OCD) (89.8 %), whilst the narrowest gap was among those with dysthymia (46.8 %) followed by major depressive disorder (MDD) (59.6 %). Of those who sought help, there was also a considerable delay in doing so: the shortest was among those with MDD (median of 4 years) and the longest was for alcohol abuse (median of 13 years) [3]. Data from the survey also showed that a majority of those who did not seek help for their mental health problems either thought that they could handle the problem by themselves (55.6 %) or did not think they had a problem (31.6 %) [4].

Help-seeking behaviour is complex, comprising multiple factors which interact with each other. Among the factors affecting help-seeking is the level of mental health literacy. The term ‘mental health literacy’ was introduced by Jorm et al. [5] who defined it as “knowledge and beliefs about mental disorders which aid their recognition, management or prevention”. A review of more than 30 national studies [6] showed that a substantial proportion of the lay public could not correctly recognize mental disorders and often attributed mental illness to psychosocial stress rather than a medical disorder. This has a number of important implications. Early and appropriate help-seeking is associated with improved long-term outcomes for those with mental disorders [7]. The failure to recognize signs and symptoms of mental disorders leads to a delay in help-seeking [8] or seeking help from inappropriate sources. In Malaysia for example, witchcraft and possession by spirits is believed to be an important cause of mental illness which in turn led people to seek help from traditional and religious healers rather than mental health professionals [9]. Furthermore, once a person decides to seek help from a care-provider, it is important that they use the appropriate ‘terminology’ to communicate with health professionals. It has been shown that General Practitioners are more likely to detect and diagnose a mental disorder if the patient conceptualizes the problem as a psychological problem [10] and when the patient explicitly raises the problem [11].

Differences in mental health literacy have been observed across different age groups and gender. Farrer et al. [12] found that younger people had more accurate knowledge than the elderly surrounding the recognition and treatment of depression. Among Australians, Cotton et al. [13] found significant gender differences in mental health literacy; males showed significantly lower recognition of symptoms associated with mental illness and were more likely to endorse the use of alcohol to deal with mental health problems.

Improving mental health literacy of which proper recognition and labeling of mental disorders form**s** an important component, is pivotal in increasing appropriate help-seeking and ameliorating the high level of unmet needs in the treatment of mental disorders [14, 15]. Given the wide treatment gaps found in Singapore with the attendant consequences of untreated illnesses, it is important to examine in greater extent and depth the possible role that poor mental health literacy may play in terms of treatment gaps so that appropriate measures can be taken.

The aim of this first population-based mental health literacy study in Singapore was to determine the proportion of adults (those aged 18–65 years) in the resident population who are able to recognize five common mental disorders (alcohol abuse, dementia, depression, schizophrenia and OCD) correctly. Sociodemographic factors associated with the ability to correctly recognize mental disorders were also explored. These disorders were selected based on a number of factors which were established by recent surveys on the mental health status of the adult and older adult population of Singapore [2, 16], including their relatively high prevalence in the population, and in particular the large treatment gap of each of these disorders.

## Methods

### Setting and study design

Singapore has a multi-ethnic population of approximately 5.5 million people in 2014, of which 3.9 million are Singapore citizens or Permanent Residents (PRs). The resident population comprises Chinese (74.3 %), Malays (13.3 %) and Indians (9.1 %), while 3.3 % are of other ethnic groups [17].

This was a nationwide cross-sectional study of adult Singapore Residents (Citizens and PRs) belonging to the four major ethnic groups, who were literate in English, Malay, Chinese or Tamil language. The study was approved by the relevant Institutional and Ethics Committees (Clinical Research Committee, Institute of Mental Health Singapore and National Healthcare Group Domain Specific Review Board, Singapore). Written informed consent was taken from all respondents who were 21 years and above as well as from parents or guardians of participants who were aged 18–20 years.

### Sampling

The study population consisted of Singapore Residents aged 18 to 65 years who were living in Singapore at the time of the survey. Residents who were living outside the country and not contactable due to incomplete or incorrect addresses were excluded from the study. A disproportionate stratified sampling design by age and ethnicity groups was implemented for the study. A probability sample was randomly selected using this sampling design with 12 strata defined accordingly to ethnicity (Chinese, Malay, Indian, Others) and age group (18–34, 35–49, 50–65). Residents aged 50–65 years, Malays and Indians were over-sampled to ensure that sufficient sample size would be achieved to improve the reliability of estimates for the subgroup analysis. The sample was derived using the sampling frame from an administrative database in Singapore that maintains the names, socio-demographic details such as age, gender, ethnicity and household addresses of all citizens, PRs and foreigners in Singapore.

### Sample size

Statistical power calculations for binary proportions after adjusting for design effect were estimated to determine the sample sizes for the overall prevalence estimate, as well as for sub-groups by age and ethnicity, with precision of 4 % [18]. The design effect after oversampling by age and ethnicity was 1.87. Using 20 % as a prevalence estimate for correct recognition of causes of mental disorders in Singapore, as previously reported in an earlier study [19], a sample size of 600 was calculated for each disorder. A total sample size of 3000 (5 disorders x 600 cases) with the margin of error was then computed. The margin of error for the overall prevalence estimate was found to be 2 %, while the margin of error for the strata defined by age and ethnicity ranged from 3.3 % to 3.5 %. Relative standard error (RSE) was calculated and ranged from 5 to 9 % and was found to be below the acceptable range of 30 % [20]. A target sample size of 3000 was thus estimated to be adequate to provide sufficient precision for the study.

### Questionnaire

Socio-demographic information on the participants was collected using a structured questionnaire that included their age, gender, ethnicity, marital, educational and employment status, and personal income. Mental health literacy was assessed using a questionnaire modeled on the Depression Literacy Questionnaire developed by Jorm et al. [5]. Permission was sought and obtained from the author for the use of the questionnaire (Jorm AF, personal communication on 2 May 2012). In accordance with the framework, respondents were presented with a vignette describing the specific disorder, and asked what, if anything, they thought was wrong with the person described in the vignette and to name the condition causing these symptoms. The response was coded as correct if the respondent was able to correctly label the specific condition. In the situation where the response was a near approximation of the correct answer, two of the investigators (MS and CSA) would come to a consensus on how that response should be coded.

### Cognitive testing, adaptation and translation of vignettes

In all, 5 vignettes were used for the study. The vignettes were developed and revised in consultation with experienced research psychiatrists and were further vetted by a panel of senior clinical psychiatrists to ensure that these vignettes satisfied the DSM-IV diagnostic criteria for each disorder (alcohol abuse, dementia, depression, schizophrenia and OCD). The case vignettes were further tested using cognitive interviews (CIs), which were completed with 75 participants who were selected to represent different age-groups, genders, ethnicity and socio-economic strata. They were instructed by trained interviewers using set protocols to read the vignettes and answer follow up questions. They were then systematically probed on what they thought the vignette was about, whether they could repeat the questions and what came to their mind when they heard a particular phrase or term. They were asked how they decided on their response. CI participants also reported any word they did not understand and any word or expression that they found offensive or unacceptable; and where alternative words or expressions exist for one item or expression, the respondent was asked which of the alternatives conforms better to their usual language. The vignettes pertaining to the study have been included in a Additional file 1.

Once the vignettes were finalized, they (together with the additional questionnaires) were translated into the three local languages – Mandarin Chinese, Malay and Tamil by a professional translating firm. The translation procedure undertaken was aimed at achieving conceptual equivalence using a four-step process that was adapted from the WHO method: 1) single forward translation, 2) expert panel review, 3) pre-testing and cognitive interviewing and 4) development of a final version.

### Data collection

Only one vignette was assigned to each respondent via a pre-determined randomization algorithm which was computer generated at the time of the interview and interviewers were blinded to this algorithm. The vignette assigned to the respondents described a person of the same gender and ethnicity as themselves. Real-time data was collected using iPAD based computer assisted personal interviews (CAPI). For quality assurance, 20 % of the completed interviews were validated through face to face and telephone follow-ups.

### Statistical analyses

All estimates were weighted to adjust for over sampling and post-stratified for age and ethnicity distributions between the survey sample and the Singapore Resident population in the year 2012. Weighted mean and standard error of the mean were calculated for continuous variables, and frequencies and percentages for categorical variables. Descriptive statistics were used to establish the prevalence of mental health literacy as well as describe the sociodemographic characteristics of the sample. Cross tabulation with Chi-square test was used to evaluate differences among gender, age groups, ethnicity and other sociodemographic variables. Multiple logistic regression analyses were carried out to determine the sociodemographic correlates of correct recognition of mental disorders. Standard errors (SE) and significance tests for survey data analysis procedures were estimated using the Taylor series’ linearisation method to adjust for the weighting. Multivariate significance was evaluated using Wald *X*^2^ tests based on design corrected coefficient variance-covariance matrices. All analyses were conducted with the SAS Software and statistical significance was evaluated at the 0.05 level using 2-sided tests.

## Results

Out of the 4,231 individuals contacted, 3,006 respondents completed the study giving a response rate of 71 %. Table 1 shows the sociodemographic distribution of the respondents. The mean age of the respondents was 40.9 years. About 50.9 % of the respondents were males. Most (74.7 %) were of Chinese descent, 12.8 % were Malays, 9.1 % were Indians, and 3.3 % belonged to other ethnic groups.

**Table 1:** Sociodemographic characteristics of the study sample

	*N*	Weighted %	SE
Age group			
18 to 34 years	1152	34.4	0.04
35 to 49 years	896	35.2	0.04
50 to 65 years	958	30.5	0.1
Gender			
Female	1506	49.1	1.3
Male	1500	50.9	1.3
Ethnicity			
Chinese	1034	74.7	0.04
Malay	977	12.8	0.01
Indian	963	9.1	0.01
Others	32	3.3	0.04
Marital status			
Married	1916	64.0	1.0
Never married	927	31.4	0.9
Others (Divorced,Widowed,Separated)	162	4.6	0.5
Education			
6 years of education and below	431	13.4	0.8
Secondary education	820	25.8	1.0
A level, polytechnic and other diploma	999	31.3	1.1
University	756	29.6	1.1
Employment status			
Employed	2227	77.6	1.0
Housewife/homemaker	378	8.7	0.6
Retired	78	3.0	0.4
Student	203	6.7	0.5
Unemployed	120	3.9	0.5
Income			
< SGD2000	1346	40.5	1.2
SGD2000-5999	1162	46.4	1.3
SGD6000 and above	2802	13.1	0.9

A level: General Certificate of Education Advanced Level, denotes completion of pre-University education

While 60.3 % (*n* = 1727) recognized the condition as a mental illness (including mislabeling of disorders and referring to them merely as a ‘mental illness’), the rate of overall correct recognition of specific conditions was 43.7 % (*n* = 1173). Table 2 shows the percentage of respondents endorsing each category in relation to recognition of the vignettes. The most well recognized vignettes were those pertaining to dementia (66.3 %), alcohol abuse (57.1 %) and depression (55.2 %). Only 11.5 % correctly recognized schizophrenia which was the most poorly recognised condition of all the five disorders. It was most commonly described as a “psychological, mental and emotional problem” (29.4 %) or was wrongly labeled by 17 % of the respondents as depression. The rate of recognition for the OCD vignette (28.7 %) was also low and most respondents had used terms that described the symptoms such as ‘cleanliness disorder’, ‘excessive fear of dirt’ which were coded as “disorder specific symptoms”.

**Table 2:** Percentage of respondents mentioning each category to describe the problem in the vignette

Category	Alcohol Abuse (*n* = 603)			Dementia (*n* = 596)			Depression (*n* = 607)			OCD (*n* = 605)			Schizophrenia (*n* = 595)		
	*n*	%	SE	*n*	%	SE	*n*	%	SE	*n*	%	SE	*n*	%	SE
Correct recognition	339	57.1	2.7	300	66.3	2.6	317	55.2	2.8	157	28.7	2.5	60	11.5	1.8
Disorder specific symptoms	3	0.9	0.6	145	18.7	2.1	14	1.7	0.6	136	26.9	2.5	92	16.2	2.0
Loneliness	2	0.1	0.1	.	.	.	5	0.2	0.1	2	0.1	0.1	53	8.8	1.6
Other mental illness –anxiety	.	.	.	1	0.05	0.05	2	0.7	0.5	28	4.3	1.1	6	0.6	0.3
Other mental illness –depression	48	5.5	1.1	10	1.2	0.6	.	.	.	7	1.2	0.6	100	17.0	2.1
Other mental illness -miscellaneous	.	.	.	2	0.1	0.1	3	0.4	0.3	20	3.2	1.0	23	3.4	1.0
Psychological/mental/emotional problem	14	1.6	0.6	15	2.3	1.2	35	4.5	1.1	78	10.4	1.6	184	29.4	2.5
Psychosocial stress	83	13.6	1.8	25	1.6	0.4	190	30.4	2.6	18	3.6	1.1	41	6.3	1.4
Not a problem	77	13.8	1.9	73	6.4	1.03	.	.	.	108	14.9	1.9	4	0.2	0.1
Others	37	7.4	1.5	25	1.6	0.4	41	6.9	1.4	51	6.7	1.3	32	6.7	1.5

Table 3 shows sociodemographic correlates of correct recognition of mental disorders by the vignettes. Multiple logistic regression analyses revealed that those who were never married (vs. married) had higher odds of recognition for alcohol abuse, while those with lower education (vs. university) had lower odds. Recognition of dementia was significantly lower among those of Indian and Malay ethnicity (vs. Chinese), those who were divorced, separated and widowed (vs. married) and those with lower education (vs. university). Recognition of depression was significantly higher among females but lower among Indians and those with 6 years of education or below.

**Table 3:** Sociodemographic correlates of correct recognition of mental disorders by the vignettes

	Alcohol abuse				Dementia				Depression				OCD				Schizophrenia			
	OR	95 % CI		*p* value	OR	95 % CI		*p* value	OR	95 % CI		*p* value	OR	95 % CI		*p* value	OR	95 % CI		*p* value
Age group																				
18 to 34 years	1				1				1				1				1			
35 to 49 years	1.1	0.6	2.3	0.729	2	0.9	4.2	0.077	0.6	0.3	1.2	0.149	0.5	0.2	1	0.058	0.9	0.3	2.5	0.887
50 to 65 years	1.6	0.7	3.5	0.28	1.02	0.4	2.5	0.973	0.8	0.4	1.8	0.607	0.2	0.1	0.7	0.008	0.9	0.3	2.7	0.872
Gender																				
Male	1				1				1				1				1			
Female	0.7	0.4	1.2	0.163	1.2	0.7	2.1	0.565	2.1	1.2	3.6	0.009	2.9	1.5	5.6	0.002	1.4	0.6	2.9	0.41
Ethnicity																				
Chinese	1				1				1				1				1			
Indian	1.1	0.6	1.8	0.842	0.1	0.1	0.2	<0.001	0.6	0.4	0.9	0.025	0.6	0.3	1.1	0.1	0.8	0.4	1.6	0.504
Malay	1.2	0.8	2	0.367	0.3	0.2	0.4	<0.001	1.0	0.6	1.6	0.994	1.3	0.7	2.3	0.449	0.6	0.3	1.4	0.229
Others	9.2	1.3	64.4	0.026	0.1	0.03	0.7	0.014	0.5	0.1	3.6	0.484	10.5	1.5	75.3	0.02	0.9	0.1	8.3	0.96
Marital status																				
Married	1				1				1				1				1			
Never married	2.4	1.2	4.7	0.013	0.8	0.4	1.6	0.489	1.5	0.8	2.9	0.225	1.5	0.7	3.5	0.298	0.8	0.3	2.1	0.681
Others*	1.5	0.4	4.8	0.543	0.2	0.1	0.9	0.028	1.5	0.5	4.3	0.488	0.5	0.1	2.4	0.402	1.4	0.3	7.1	0.662
Education																				
University	1				1				1				1				1			
6 years of education or below	0.1	0.03	0.2	<0.001	0.1	0.03	0.3	<0.001	0.2	0.1	0.7	0.009	0.1	0.01	0.4	0.004	0.3	0.04	2.6	0.28
Secondary	0.3	0.1	0.6	0.001	0.2	0.1	0.6	0.001	0.7	0.3	1.5	0.358	0.04	0.01	0.2	<0.001	0.6	0.2	1.9	0.418
A Level, polytechnic & other diploma	0.4	0.2	0.8	0.017	0.2	0.1	0.4	<0.001	0.6	0.3	1.1	0.112	0.4	0.2	0.9	0.034	1.1	0.4	3	0.792
Employment status																				
Employed	1				1				1				1				1			
Housewife	0.9	0.4	2.4	0.878	0.6	0.2	2	0.385	0.7	0.2	1.8	0.431	3.1	0.6	15.8	0.165	1.2	0.3	4.9	0.833
Retired	0.6	0.1	2.6	0.465	1.6	0.3	10.2	0.612	0.3	0.1	1.6	0.166	.	.	.	.	.	.	.	.
Student	1.0	0.3	4.2	0.961	2.5	0.8	8.4	0.128	2.6	0.7	9.6	0.141	2.1	0.6	6.8	0.224	1	0.1	7.8	0.988
Unemployed	0.8	0.2	3	0.704	1.2	0.3	4.4	0.791	2.2	0.5	9.7	0.31	3.4	0.7	15.4	0.117	1.6	0.4	7.6	0.529
Income																				
<SGD2000	1				1				1				1				1			
SGD2000-5999	1.0	0.5	1.8	0.995	0.8	0.4	1.6	0.53	1.1	0.6	2.2	0.689	1.4	0.6	3.1	0.439	0.7	0.3	1.8	0.452
SGD6000 & above	1.9	0.6	6.4	0.291	1.1	0.4	3	0.935	1.5	0.5	4.3	0.496	2.1	0.5	8.3	0.279	1.7	0.4	6.8	0.478

Others* - Divorced, Separated and Widowed

We found that those of older age (50 to 65 years vs 18 to 34 years) and those with lower education status had significantly lower odds of recognition for OCD while female gender had higher odds. There were no statistical significant differences found in terms of the schizophrenia vignette.

## Discussion

This national study showed that less than half of the adult population of Singapore could correctly recognize one of the five common mental disorders. The most commonly recognized of the five disorders was dementia. Singapore has one of the fastest ageing populations in the world. The government has been cognizant of this demographic shift and has been proactive in addressing the associated emerging public health, social and economic challenges. A slew of measures have been implemented including public education initiatives [21, 22]. In addition, a recent nation-wide epidemiological study among those aged 60 years and above established the prevalence of dementia to be 10 % [16] which in turn, has generated a considerable amount of interest in the mass media and the general public. The relatively greater recognition of dementia could be a consequence of these concerted national efforts and developments.

Slightly more than half of the respondents could also recognize alcohol abuse and depression. These proportions are considerably more than those correctly recognising OCD and schizophrenia. We are unable to offer any definite explanation; we think that these differences are not related to the prevalence of the disorders in the population: the prevalence of both alcohol abuse and OCD is about 3 % in Singapore’s population [2] but the rates of their recognition were significantly different. Alcohol-related problems, as well as depression, have been more extensively portrayed in popular media including television, movies, print and social media which have also seen well-known personalities and celebrities admitting to depression and alcohol-related problems as compared to OCD and schizophrenia which might have resulted in the higher recognition rates of the same.

In a study conducted in Shanghai (China), it was found that only about 8 % of the participants correctly attached a schizophrenia and psychosis label to the vignette [23]. The authors cited two possible reasons. The first was the great degree of stigmatization of mental illness and the prevalent traditional beliefs that framed mental illness as the effect of “being possessed by demons; improper child bearing practice; or the wrongdoing of one’s ancestors”. The second was the lack of public education on mental health and mental illness.

Similarly in this study, schizophrenia is the least recognized disorder where only 11.5 % if the respondents correctly identified the condition. This is even more startling when compared to 42.5 % of the Australian adult public who could correctly recognise this disorder via a vignette, in 2003–2004 [24]. Even though schizophrenia may be a relatively low prevalence disorder – there has yet to be a population-based study to determine its prevalence in Singapore – this lack of recognition is of concern due to the severity and chronicity of this disorder and enormous impact on the individual, family and society.

The recognition of OCD was also relatively poor in Singapore, similar to a community survey from the United States which showed that only one-third of the participants could correctly recognize OCD [25]. The prevalence of OCD in Singapore was 3.0 % - a rate that is higher than those reported in other countries and it was associated with a significant treatment gap [26]. Thus, efforts to improve literacy of OCD using educational interventions need to be considered.

While depression was relatively well recognized, there was at the same time, a mislabeling of schizophrenia as depression by 17 % of the respondents who received the vignette on schizophrenia. This phenomenon was also observed among the Australian public where a 2011 survey found the use of the label “depression” to be “over-generalized” in that it was also commonly used to describe the other disorders such schizophrenia, social phobia, and post-traumatic stress disorder [27]. The authors of this paper posited that while the use of depression across a range of disorders might be helpful in prompting professional help-seeking, they also emphasized the need to build on public knowledge of depression in order to differentiate its symptoms and treatments from other disorders. Related to this point, a third of the respondents in this study who had received the depression vignette construed the problem to be “psychosocial stress”.

In an earlier study, we have found that the treatment gap for MDD was the narrowest when compared to alcohol abuse which has the largest treatment gap of 96 % and OCD with a treatment gap of 90 % [3]. With regards to the mental health literacy of the three disorders as a possible mediator for help-seeking, it appears consistent with the level of correct recognition which was relatively high for depression and low for OCD. However, it was surprising that despite a high recognition rate for alcohol abuse, the treatment gap was so vast. It is possible that recognition of alcohol abuse might not equate to seeing a need for professional help – fostered by societal tolerance even in cases of excessive use of alcohol [28]. It may be perceived as a social ill rather than a medical problem that needs treatment. Studies demonstrating effectiveness of interventions are limited [29] and people may thus have low expectations from treatment.

Gender is another factor that may influence mental health literacy. Gender differences in physical and mental health occur with respect to illness incidence, prognosis, morbidity and mortality [30–32]. Differences have also been found with respect to perceptions and awareness of illness. In general, females tend to be more aware of symptoms than men who tend to be unaware of health problems and are more likely to delay seeking help [31, 33]. The finding of this study with regards to gender is consistent with the body of extant literature: we found that females were specifically better able to recognize depression and OCD. A similar difference was observed among non-medical students in Malaysia where female respondents had better knowledge of the symptoms of depression [34]. Similarly, another Australian study demonstrated that males had poorer mental health literacy than females with respect to depression [35].

Higher levels of education were also associated with better mental health literacy for dementia, depression, alcohol abuse, and OCD. This could be due to the acquisition of some aspects of mental health literacy through the educational system and process, as well as having better access to mental health information and being able to perceive the relevance of such information that comes from being better educated.

Surveys of mental health literacy conducted in a few countries showed significant ethnic differences in how mental illnesses are explained and labelled. Hong Kong Chinese were more likely to believe in social factors as the cause of schizophrenia as compared to the English in England who were more likely to endorse genetic factors [36]; and Japanese were less likely to use psychiatric labels as compared to Australians [37]. In this study, we found ethnicity to be a factor associated with mental health literacy for some disorders – Indians were less able to identify depression and dementia, and Malays were less likely to recognize dementia. They tended to consider it ‘not a disorder’ or used disorder specific symptoms like ‘memory loss’ and ‘senility’ to describe the disorder. This could be due to the tendency of certain ethnic groups to “normalize” or attribute the manifestations of certain mental disorders to causes other than a disease state. However, whether these are underlying reasons for the ethnic differences in mental health literacy remains to be elucidated.

The strengths of the study include the fact that it was a nationwide study using a representative sample and had a good response rate (71 %). Vignettes were constructed using inputs from psychiatrists who are experts in the field and cognitively tested before use in the survey. Vignettes and questionnaires were translated into the local languages ensuring inclusivity. However, the results of the study must be interpreted while keeping some of the limitations in perspective. While the study had a reasonably good response rate, 29 % of the sample identified did not take part in the study. It is possible that the mental health literacy of this group is significantly different from that of those assessed and it may affect the overall results. Data on the age and ethnicity of the non-respondents was available, but not on their educational status, which turned out to be a significant variable in this study. It is therefore difficult to predict how the non-response rate could have affected the study results. Another limitation pertains to the vignettes which described persons with prototypical and severe symptoms; vignettes with more subtle or uncommon symptoms may not elicit similar results. It is also possible that the random assignment of the vignettes that controlled for age and ethnicity resulted in a biased distribution in terms of other relevant variables such as the type of occupation (particularly mental healthcare providers) among the respondents which may have resulted in higher recognition in a group administered a specific vignette. While we did not collect detailed information on the occupational status of the respondents, the study included a question on whether they had ever had a job that involved providing treatment or services to a person with a problem like the respondent. Analysis of the responses did not reveal a significant difference across vignettes with affirmative responses ranging from 4.2 % for dementia to 5.6 % for depression.

## Conclusion

The findings of this study indicate the need for more extensive efforts to raise mental health literacy among the local population, particularly for schizophrenia and OCD. The study has also identified certain subgroups that possess poorer mental health literacy including males, those of lower education, and the Indian population in Singapore who have lower rates of recognition for depression even though the rate of MDD was higher among the Indians as compared to the Chinese and Malays [38]. Additional research is needed to ascertain why such ethnic and gender differences in knowledge and perception about mental illness exist. Consideration needs to be given to the sort of information and educational strategies that would more effectively target the mental health literacy of males and those of lower educational levels, while using culturally appropriate approaches for certain ethnic groups. Further research is also need to explicate the link between mental health literacy and the actual and appropriate help-seeking behaviour.

### Ethics and consent to participate

The study was approved by the National Healthcare Group Domain Specific Review Board in Singapore and written informed consent was obtained from the participants.

### Consent to publish

Not applicable

### Availability of data and materials

Data is not available for online access, however readers who wish to gain access to the data can write to the corresponding author Dr Mythily Subramaniam at mythily@imh.com.sg with their requests. Access can be granted subject to the Institutional Review Board (IRB) and the research collaborative agreement guidelines. This is a requirement mandated for this research study by our IRB and funders.

## Additional file

Additional file 1: All the vignettes administered in this study are available in the supplementary file. (DOCX 18 kb)

## Abbreviations

CAPI: computer assisted personal interviews
CI: cognitive interview
MDD: major depressive disorder
OCD: obsessive compulsive disorder
PR: permanent resident
RSE: relative standard error
SE: standard error
WHO: World Health Organization
