# Supplementary material for: Recognition of mental disorders among a multiracial population in Southeast Asia
Source: BMC Psychiatry. 2016 May 4;16:121. doi: 10.1186/s12888-016-0837-2 (PMC4855433; doi:10.1186/s12888-016-0837-2)
Supplement: Additional file 1: — All the vignettes administered in this study are available in the supplementary file. (DOCX 18 kb) [file 12888_2016_837_MOESM1_ESM.docx]

**Vignettes used in this study:**

Depression- XX is 30 years old. He has been feeling unusually sad and miserable for the last three weeks. Friends noticed he is no longer his usual cheerful self and he has declined all social gatherings over the past two weeks. Even though he is tired all the time, he has trouble sleeping almost every night. XX doesn't feel like eating and has lost weight. He can't focus on his work and puts off making decisions. XX feels worthless and even everyday tasks seem too much for him. This has come to the attention of his boss, who is concerned about XX's poor work performance.

Schizophrenia- XX (insert name) is 44 years old. He is staying in a 1-room HDB rental flat. He has not worked for years. He wears the same clothes every day and has left his hair to grow long and untidy. He is always on his own and is often seen sitting in the park talking to himself. Sometimes he stands and moves his hands as if to communicate to someone in nearby trees. He rarely drinks alcohol. At times he accuses shopkeepers of giving information about him to other people. He has put extra locks on his door. He says spies are watching him all the time. His neighbors complain that he does not clean his room which is becoming increasingly dirty and is filled with glass objects. XX says he is using these "to receive messages from space".

OCD- XX (insert name) is 37 years old and each day he spends 4 hours washing his hands. He usually takes one shower a day but he spends 60–90 min in the shower. When XX washes his hair he keeps the shampoo in his hair until he has counted to 100 to ensure that his head and hair are clean enough and free of contaminants, such as germs. XX also repeatedly cleans things he touches, including dishes, clothes, furniture, and doorknobs. XX feels extremely anxious if he does not wash his hands or cleans things he touches and finds it difficult to stop himself from doing these things. .

Alcohol abuse- XX (insert name) started drinking when he was a student. He was very popular at parties. By the time he had graduated and got married he was drinking heavily every weekend. This sometimes resulted in him getting into fights when he was out drinking. Although his wife insisted that he drank too much, XX argued that he was in control. But his work and appearance deteriorated so much that his supervisor began to suspect that he might be drinking on the job. A few months later he was involved in a serious car accident, where he crashed into two cars, seriously damaging them and his own car. The police who arrived at the scene of the accident did a breathalyzer test. The test turned out to be positive and so they took his blood for alcohol analysis. As his alcohol level was much higher than the legal limit he was charged with drunk driving.

Dementia- XX is 75 years old and retired. His wife has noticed that he has problems remembering things that happened recently but recalls things from earlier in their marriage quite well. He repeats questions which she has already answered, misplaces his things and occasionally gets confused during their conversations. Sometimes XX and his wife quarrel as he accuses her of taking his things. He lost his way once or twice whilst driving to their son’s home, and has written some cheques for the wrong amount when paying bills. When his wife points out these problems to XX, he loses his temper. He does not think he has a problem.
